# Evaluation of colostrum as an alternative biological sample for the diagnosis of human congenital toxoplasmosis

**DOI:** 10.1186/s12879-015-1242-z

**Published:** 2015-11-14

**Authors:** Ana Carolina de Morais Oliveira, Hellen Dayane Silva Borges, Fernando Reis Carvalho, Arlindo Gomes de Macêdo, Caroline Martins Mota, Angela Maria de Morais Oliveira, Fernanda Maria Santiago, Cristina Guimarães Arantes Araújo, Deise Aparecida de Oliveira Silva, Tiago Wilson Patriarca Mineo, Vânia Olivetti Steffen Abdallah, José Roberto Mineo

**Affiliations:** Laboratório de Imunoparasitologia “Dr. Mário Endsfeldz Camargo”, Instituto de Ciências Biomédicas, Universidade Federal de Uberlândia (UFU), Campus Umuarama, 38400-902 Uberlândia, MG Brazil; Banco de Leite Humano do Hospital de Clínicas da Faculdade de Medicina da UFU, Campus Umuarama, Uberlândia, MG Brazil; Departamento de Pediatria do Hospital de Clínicas, Faculdade de Medicina da UFU, Campus Umuarama, Uberlândia, MG Brazil; Laboratório de Ensino da Biomedicina, Instituto de Ciências da Saúde, Universidade Federal do Triângulo Mineiro, 38025-180 Uberaba, MG Brazil

**Keywords:** *Toxoplasma gondii*, Antibodies, Colostrum, Congenital toxoplasmosis, Diagnosis

## Abstract

**Background:**

Toxoplasmosis is a zoonosis caused by *Toxoplasma gondii,* an intracellular protozoan parasite able to infect a wide range of hosts, including humans. Congenital infection can cause severe damage to the fetus. Thus, it is important to detect antibodies against the parasite to confirm clinical manifestations. Considering that all immunoglobulin isotypes may be present in biological samples from newborns and their mothers, this study aimed to evaluate the ability to diagnose recent toxoplasmosis by using colostrum, as an alternative noninvasive way to obtain biological samples, as well as to determine correlation rates between antibodies from serum samples to detect IgG, IgM and IgA isotypes against *T. gondii*.

**Methods:**

A total of 289 puerperal women from Clinical Hospital of Federal University of Uberlândia (mean age: 24.8 years, range: 14 – 43 years) took part in this study. Serum and colostrum samples from these patients were analyzed using ELISA and immunoblotting assays for soluble antigens from *T. gondii.*

**Results:**

ELISA immunoassays with serum samples showed reactivity in 47.0, 6.9 and 2.8 % of samples to anti*-T. gondii* IgG, IgM and IgA, respectively, in comparison with colostrum samples, which showed reactivity in 46.0, 7.9 and 2.8 % of samples to the same isotypes. Also, significant correlation rates of anti-*T. gondii* antibody levels between serum and colostrum samples were observed. Interestingly, reactivity to IgM and/or IgA in colostrum and/or serum confirmed clinical manifestations of congenital toxoplasmosis in three newborns. Immunoblotting assays showed that it is possible to detect IgG, IgM and IgA antibodies against various antigens of *T. gondii* in serum and colostrum samples. IgG antibodies in serum and colostrum samples recognized more antigenic fractions than IgM and IgA antibodies. Serum IgG detected more antigenic fractions than IgG antibodies present in the colostrum of the same patient. In contrast, specific IgA present in colostrum recognized a higher number of antigens than IgA present in serum samples of the same patient.

**Conclusions:**

Overall, the results show that it is important to investigate the occurrence of congenital toxoplasmosis, even at puerperal period. Furthermore, this study demonstrates that *T. gondii-*specific IgG, IgM and IgA antibodies in serum and colostrum samples from puerperal women may be detected with a significant correlation, suggesting that colostrum may also be used as an alternative biological sample to efficiently diagnose recent human toxoplasmosis.

## Background

Toxoplasmosis is an infectious disease caused by *Toxoplasma gondii,* an obligate intracellular protozoan parasite that infects different hosts, including a third of the human population worldwide [1, 2]. The infection is acquired mainly by ingestion of water or food contaminated with oocysts shed in felidae feces, by ingestion of raw meat containing tissue cysts or by congenital transference of the parasites [3]. Although, most infections are asymptomatic in immunocompetent adults, they may be fatal for immunocompromised patients and cause severe damage to the fetus affected by congenital infection [4]. The immune response against *T. gondii* involves five isotypes of human antibodies (IgG, IgM, IgA, IgE and IgD), specific to *T. gondii,* which may be detected in various biological fluids, such as serum, cerebrospinal fluid, and human colostrum or milk [5, 6].

There are several pieces of evidences showing that breastfeeding protects the infant against a wide range of infectious diseases [7–10] and there are currently many studies on human milk composition to understand the role of its bioactive components [10]. However, few efforts have been directed to demonstrate the role of human colostrum against parasitic infections, including in *T. gondii* infection, as well as its use in immunodiagnosis [6].

Considering high prevalence of toxoplasmosis, and the importance to characterize the disease in its early stage, the present study was conducted to detect the correlation of IgG, IgM and IgA antibodies with *T. gondii* in paired samples of serum and colostrum, and to evaluate the ability to diagnose this disease by using colostrum, as an alternative noninvasive way to obtain biological samples.

## Methods

### Subjects

A total of 289 puerperal women participated in this study. They were patients at the Obstetric Center of the Hospital de Clínicas da Universidade Federal de Uberlândia (UFU), Minas Gerais, Brazil, with mean age 24 years, median age 24.8 years, ranging from 14 to 43 years. The study exclusion criteria consisted of HIV and/or HTLV seropositive women, in whom breastfeeding is contra-indicated by the Brazilian Health Ministry [11]. The study conformed to the declaration of Helsinki, with its protocol approved by UFU Ethical Committee (Protocol number: 104.110). A written consent was obtained from each patient.

### Biological samples

Human breast milk samples, volumes of 3 mL, were collected between the 1st and the 3rd day postpartum, to ensure that it was colostrum. The samples were centrifuged at 500 × *g* for 10 min at 4 °C to remove fat and cleared colostrum samples were stored at −20 °C. Serum samples were obtained from the patients one day after delivery and the aliquots were stored at −20 °C.

### Parasites and antigens

*T. gondii* RH strain tachyzoites were maintained by serial passage in cell culture for 48–72 h, using HeLa cell lines (ATCC/CCL-2; American Type Culture Collection, Manassas, VA, USA), as described above [12]. Briefly, cells were inoculated with tachyzoites of *T. gondii* and maintained by serial passages in RPMI medium with 2 % fetal bovine serum. Free parasites were collected and washed with phosphate buffered saline (PBS, pH 7.2) and, after addition of a protease inhibitor cocktail (Complete Ultra tablets, Roche, USA), lysates were obtained by repeated freezing and thawing cycles, sonication, and centrifugation at 14,000 × *g* for 30 min at 4 °C. After supernatant recovery, total protein was estimated by the Bicinchoninic acid kit (BCA, Sigma, St. Louis, MO, USA) and the aliquots were stored as soluble tachyzoite antigen (STAg) at −80 °C until later use.

### Immunoassays for detection of antibodies anti-*Toxoplasma gondii*

The presence of *Toxoplasma-*specific IgG, IgM and IgA antibodies was investigated in serum and colostrum samples from puerperal women by using the Enzyme Linked Immunosorbent Assay (ELISA) and Immunobloting assays.

The optimal dilutions for serum and colostrum samples in each assay, as well as the time of incubation and blocking conditions for these samples, were obtained by block-titration of the reagents.

#### Indirect ELISA to detect anti- *T. gondii* IgG antibodies

ELISA to detect IgG antibodies was carried out according to the conditions described previously [13] with modifications. Briefly, wells of high affinity microtiter plates (Costar Corning Incorporated, USA) were coated with 50 μL of STAg (10 μg/mL) in 0.06 M carbonate buffer (pH 9.6) and incubated overnight at 4 °C. Plates were washed 3 times with PBS-Tween 0.05 % (PBS-T) and blocked with PBS-T plus 5 % of nonfat powdered milk (Molico, Nestlé, São Paulo, Brazil) (PBS-T-M5 %) for 1 h at room temperature. Samples of serum (1:64 in PBS-T-M5 %) and colostrum (1:5 in PBS-T) were incubated for 1 h at 37 °C. After washing, plates were incubated with goat anti-human IgG antibody labeled with peroxidase (Sigma) for 1 h at 37 °C. The reactions were revealed by adding peroxidase substrate system (ABTS, KPL, Kirkegaard & Perry Laboratories), and optical densities (OD) were determined at 405 nm. Two positive controls and three negative controls were included in each plate in order to calculate the cut-off, which was established as the mean OD values for negative controls plus three standard deviations. Results were expressed as ELISA index (EI), as follows: EI = OD_sample_/OD_cut-off_, where values of EI ≥ 1.2 were considered positive [14].

#### Capture ELISA to detect anti- *T. gondii* IgM and IgA antibodies

ELISA to detect IgM and IgA antibodies was carried out as described previously [13] with modifications. Briefly, high affinity microtiter plates wells were coated with either human anti-IgM (KPL, Kirkegaard & Perry Laboratories) or human anti-IgA (Sigma) in 0.06 M carbonate buffer (pH 9.6) and incubated overnight at 4 °C. Plates were blocked with PBS-T-M5 % for 1 h at room temperature. Samples of serum (1:16 in PBS-T-M5 %) and colostrum (1:5 in PBS-T) were incubated for 2 h at 37 °C. After washing, plates were incubated with STAg (100 μg/mL) diluted with PBS-T-M5 %, for 2 h at 37 °C, followed by addition of rabbit anti-*T. gondii* IgG labeled with peroxidase according to Wilson and Nakane [15]. The next steps were carried out as described above.

#### Immunoblotting assays to detect anti- *T. gondii* antibodies

Immunoblotting assays were carried out to analyze the specificity and pattern recognition of serum and colostrum antibodies against STAg, as described above [16]. Briefly, STAg was separated on 12 % SDS-PAGE under non-reducing conditions, and electrotransferred to nitrocellulose membranes. Non-specific interactions were blocked by PBS-T-M5 % incubation, for 2 h at room temperature. Nitrocellulose strips were incubated with serum samples (diluted 1:100 [IgG] or 1:50 [IgM and IgA] in PBS-T plus 1 % of nonfat milk – PBS-T-M1 %) or colostrum (diluted 1:10 [IgG] or 1:5 [IgM and IgA] in PBS-T). Protein-antibody complex was detected by incubation with secondary antibodies, anti-IgG, anti-IgM or anti-IgA labelled with peroxidase (Sigma) for 2 h at room temperature. Reactions were revealed by adding diaminobenzidine (Stable DAB, Invitrogen), and stopped with distilled water. The molecular weight (KDa) for each antigenic fraction was determined using the Image Lab program Version 4.0.1 (Bio-Rad Laboratories, Hercules CA).

#### IgG avidity immunobloting assays

After incubation with serum or colostrum samples in duplicated nitrocellulose strips, one strip was washed with PBS-T (urea^−^) and the other washed with PBS-T containing 6 M of urea (urea^+^) for 10 min. After washing with PBS-T, the strips were submitted to the immunoblotting assays as described above. The intensity pixels (Int Band) obtained for each antigenic fraction of samples were evaluated using Image Lab program Version 4.0.1 (Bio-Rad Laboratories). The results for urea^−^ or urea^+^ strips were expressed in avidity index (AI), as follows: AI = (urea^+^ Int Band/urea^−^ Int Band) × 100. AI <50 % corresponds to reactivity with antibodies of low avidity, while AI > 75 % corresponds to reactivity with antibodies of high avidity.

### Statistical analysis

Statistical analysis was carried out using GraphPad Prism 5.0 (GraphPad Software Inc., San Diego, CA). Differences between levels of the same antibody isotypes in human serum and colostrum samples were analyzed using Wilcoxon test. Spearman correlation test was used to correlate the levels between the same antibody isotype in different samples or between different antibody isotypes in the same sample. Values of *p* < 0.05 were considered statistically significant.

## Results

### Detection of antibodies in serum and colostrum samples from puerperal women

*T. gondii-*specific IgG, IgM and IgA antibodies were detected by ELISA in 136 (47.0 %), 20 (6.9 %), 8 (2.8 %) serum samples and in 133 (46.0 %), 23 (7.9 %), 8 (2.8 %) colostrum samples.

**Fig. 1:**
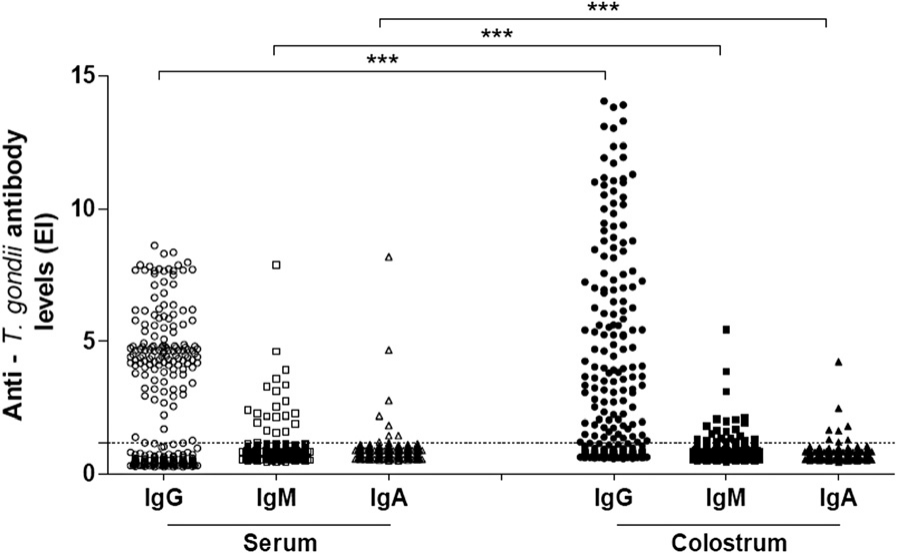
Measurement of anti-*T. gondii* antibody levels from assorted isotypes. Levels of IgG, IgM and IgA isotypes directed to the parasite were detected in serum and colostrum samples from 289 puerperal women, as determined by ELISA indices (EI). The dashed line represents the cut-off value (EI = 1.2). *** Data statistically significant (*p* < 0.0001, Wilcoxon)

The levels of anti-*T. gondii* IgG, IgM and IgA antibodies in serum and colostrum from all puerperal women participating in this study are shown in Fig. 1. The levels of IgG antibodies detected in colostrum samples (mean: 5.90; median/range [med]: 5.26/1.22–14.05) were significantly higher than IgG detected in serum samples (mean: 4.99; med: 4.66/1.28–8.62), *p* < 0.0001. In contrast, the levels of IgM and IgA antibodies detected in colostrum samples (mean: 1.91; med: 1.61/1.22–5.46 and mean: 1.96, med: 1.65/1.22–4.23, respectively) were significantly lower than IgM and IgA detected in serum samples (mean: 2.87; med: 2.36/1.58–7.88 and mean: 2.98; med: 2.02/1.22–8.20, respectively), *p* < 0.0001.

### Correlation between anti-*T. gondii* antibody levels in serum and colostrum

**Fig. 2:**
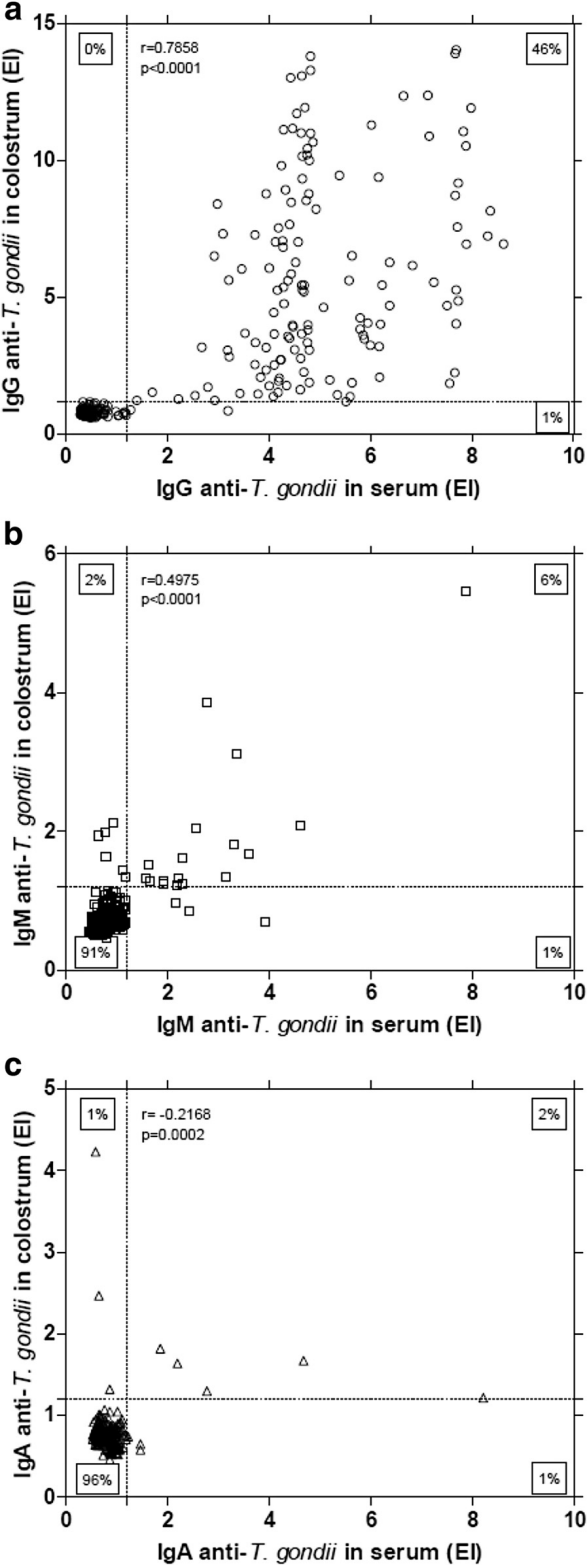
Correlation between levels of anti-*T. gondii* antibody from assorted isotypes. Levels of IgG (**a**), IgM (**b**) and IgA (**c**) antibody isotypes were compared in serum and colostrum samples from 289 puerperal women. Correlation coefficients were calculated by Spearman correlation test

When analyzing the anti-*T. gondii* antibody isotype levels detected in serum and colostrum samples, a significant positive correlation was found when the levels of IgG were compared (*r* = 0.7858; *p* < 0.0001) (Fig. 2a). A significant positive correlation was also observed when the levels of IgM were compared (*r* = 0.4975; *p* < 0.0001) (Fig. 2b), whereas, a negative correlation was found when the levels of IgA were compared (*r* = −0.2168; *p* = 0.0002) (Fig. 2c).

**Fig. 3:**
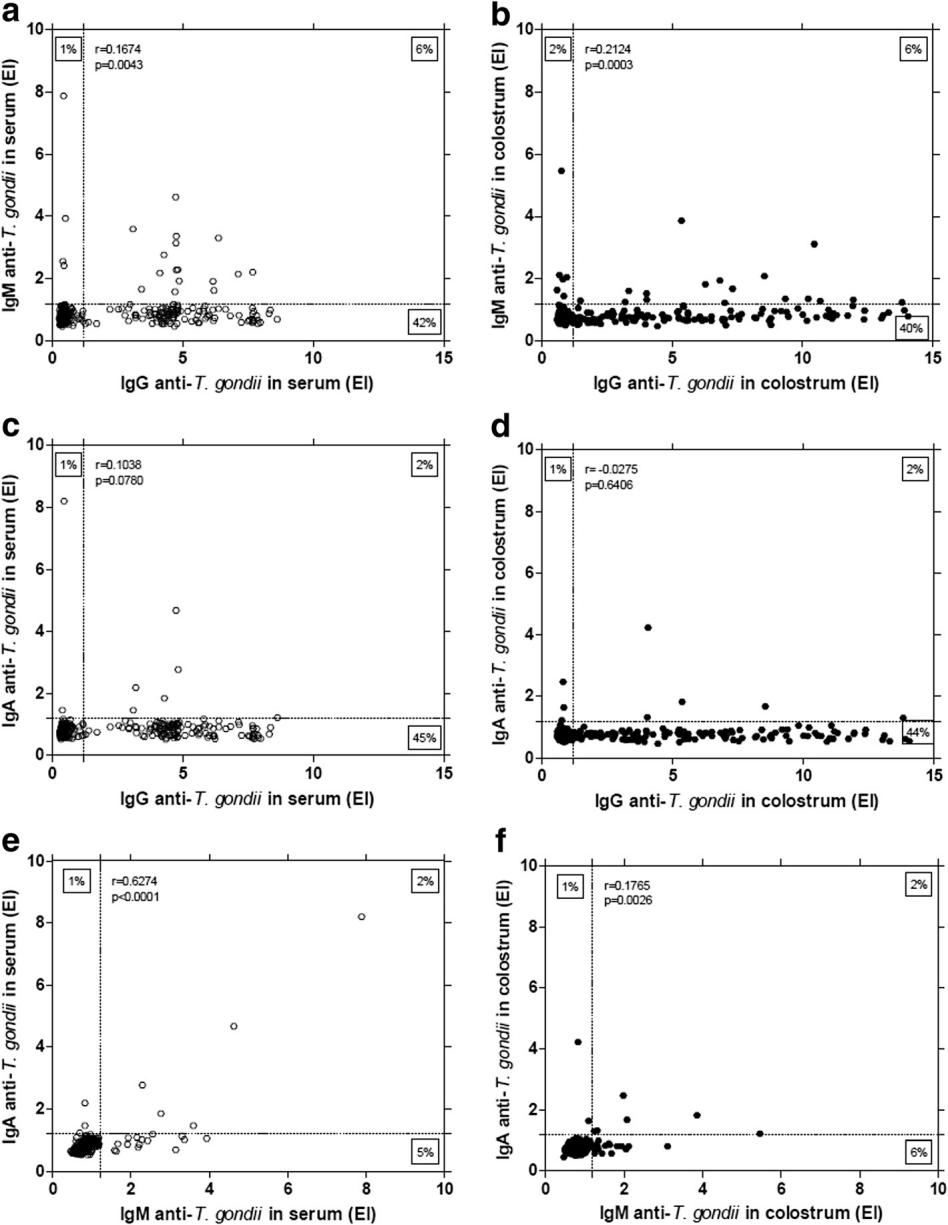
Correlation between levels of *T. gondii*-specific antibody isotypes. Samples from serum (**a**, **c**, **e**) or colostrum (**b**, **d**, **f**) were obtained from 289 puerperal women. Correlation coefficients were calculated by Spearman correlation test

The antibody isotype levels detected in serum samples showed different degrees of positive correlation, as follows: IgG *vs* IgM, low correlation, though it was statistically significant (*r* = 0.1674; *p* = 0.0043) (Fig. 3a); IgG *vs* IgA, low correlation with no statistical significance (*r* = 0.1038; *p* = 0.0780) (Fig. 3c); IgM *vs* IgA, significant correlation (*r* = 0.6274; *p* < 0.0001) (Fig. 3e). Regarding the levels of anti-*T. gondii* antibody isotypes detected in colostrum samples, positive correlations were found for IgG *vs* IgM (*r* = 0.2124; *p* = 0.0003) (Fig. 3b) and IgM *vs* IgA (*r* = 0.1765; *p* = 0.0026) (Fig. 3f).

### Associations among *T. gondii-*specific IgG, IgM and IgA in serum and colostrum

Four serum samples and four colostrum samples presented simultaneous reactivity to *T. gondii* for all three analyzed isotypes, with three samples from concordant patients (60.0 %). Simultaneous reactivity to IgG plus IgM of two isotypes was observed in serum and colostrum (4.2 and 4.5 %, respectively), to IgG plus IgA in serum and colostrum (0.7 and 0.3 %, respectively), and to IgM plus IgA in serum and colostrum samples (0.3 and 0.7 %, respectively), with 66.7 % of concordant patients to IgG plus IgM. The most frequent event was the reactivity to IgG (40.8 % in serum and 39.8 % in colostrum) with 94.2 % of concordant patients. As observed in Table 1, 94.1 % of patients had concordant results for anti-*T. gondii* antibodies when colostrum and serum samples were analyzed.

**Table 1:** Detection of *T. gondii-*specific IgG, IgM and IgA isotypes in human serum and colostrum samples from puerperal women, measured by ELISA

IgG/IgM/IgA	Serum^a^ n (%)	Colostrum^a^ n (%)	Concordant patients in serum and colostrum^a^ n (%)
+/+/+	4 (1.4)	4 (1.4)	3 (60.0)
+/+/-	12 (4.2)	13 (4.5)	10 (66.7)
+/-/+	2 (0.7)	1 (0.3)	0 (0)
-/+/+	1 (0.3)	2 (0.7)	1 (50.0)
+/-/-	118 (40.8)	115 (39.8)	113 (94.2)
-/+/-	3 (1.0)	4 (1.4)	1 (16.7)
-/-/+	1 (0.3)	1 (0.3)	0 (0)
-/-/-	148 (51.3)	149 (51.6)	144 (94.1)
Total	289 (100)	289 (100)	272 (94.1)

^a^Data are presented in number (n) and percentage (%) of the analyzed samples

### Detection of *T. gondii* antigenic fractions by specific IgG, IgM and IgA antibodies

According to the conventional serologic ELISA results, 30 patients were characterized as a risk group of acute toxoplasmosis since their samples presented IgA and/or IgM in serum and/or in colostrum associated or not to presence of IgG. Their samples were selected for immunoblotting assays in order to evaluate the detection of the three isotypes of antibodies against various *T. gondii* soluble antigens. Samples from one of these patients were not tested due to insufficient volume.

**Fig. 4:**
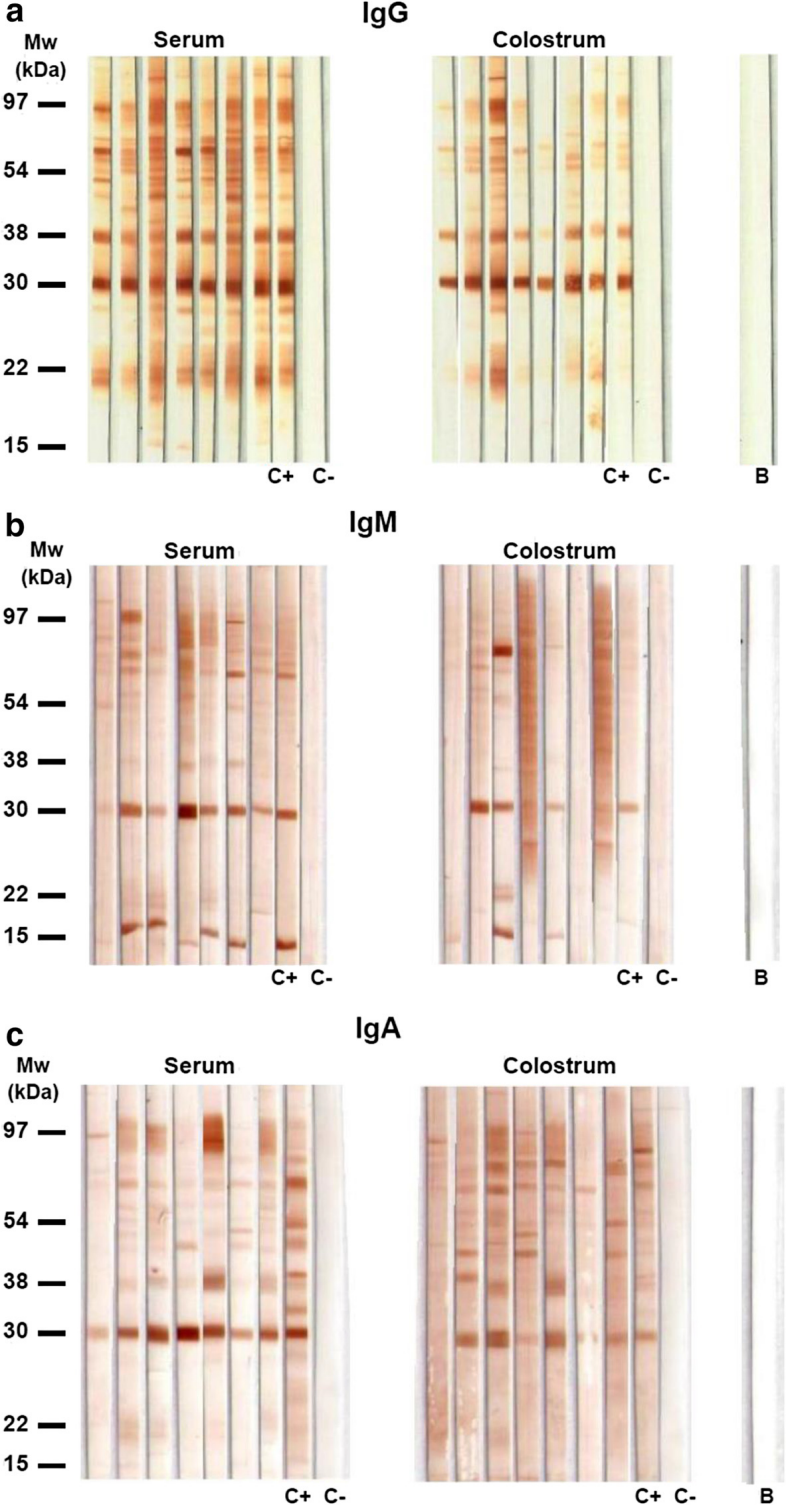
Immunoblotting results for antigenic fractions from *T. gondii*. The assay was performed with soluble antigen of *T. gondii* for detection of IgG (**a**), IgM (**b**) and IgA (**c**) isotypes directed to the parasites in serum and colostrum samples from puerperal women. Molecular weights (kDa) are shown at left side. **C+** positive control. **C-** negative control. **B** blank

**Fig. 5:**
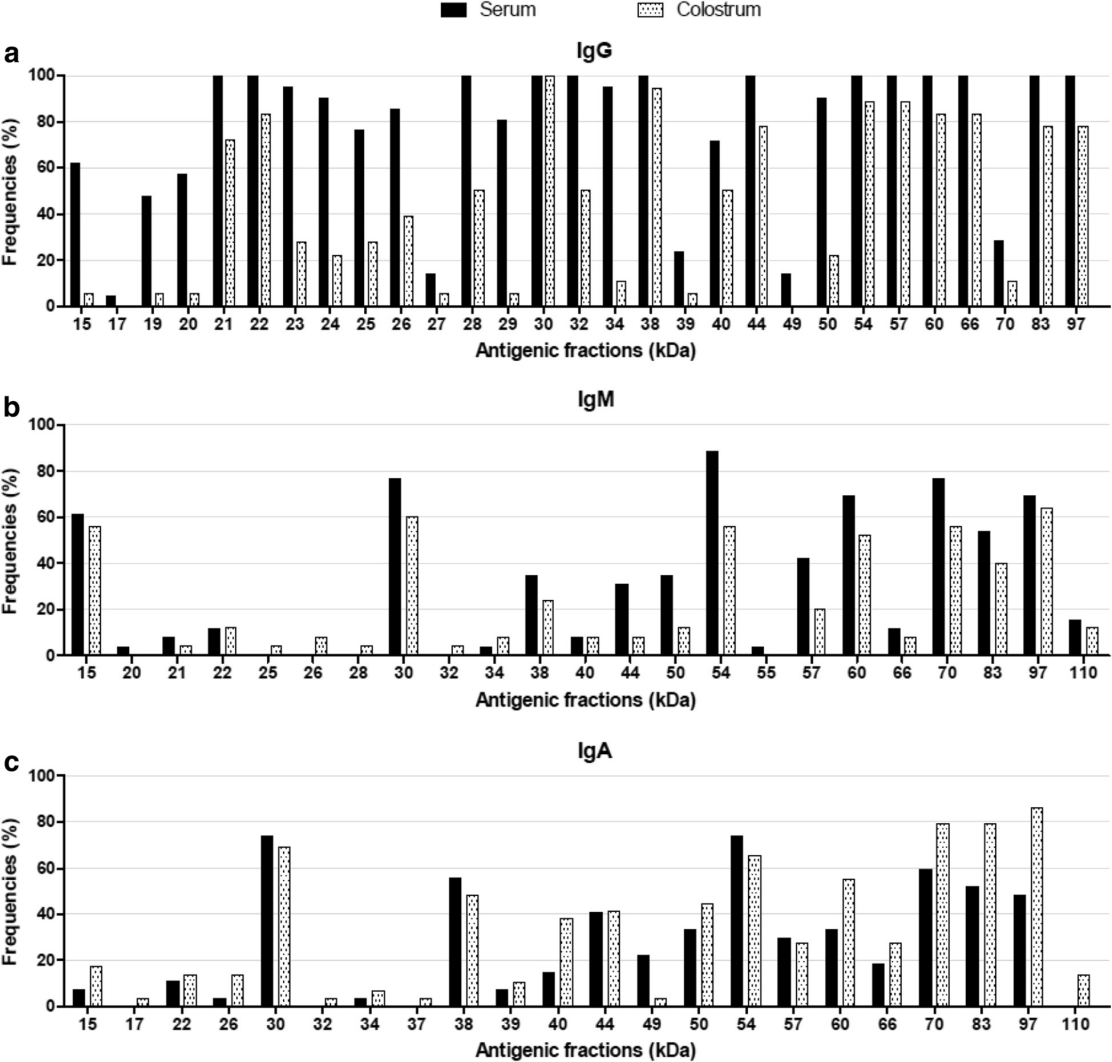
Reactivity of antibody isotypes towards antigenic fractions from *T. gondii.* Frequencies (%) of positivity for IgG (**a**), IgM (**b**) and IgA (**c**) in serum and colostrum samples that recognize each antigenic fraction (kDa) of *T. gondii*

**Fig. 6:**
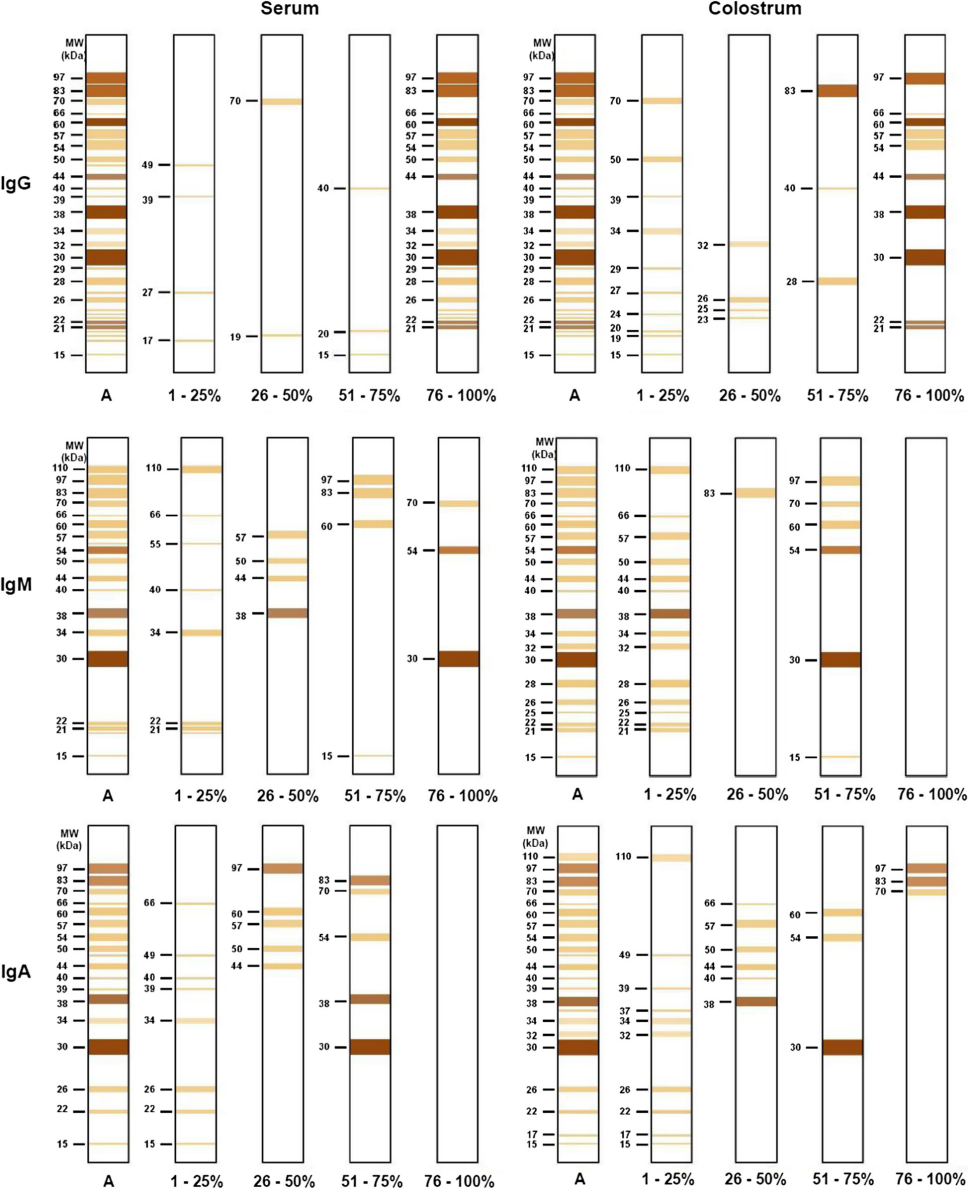
Reactivity pattern of antigenic fractions recognized by IgG, IgM and IgA antibody isotypes. Immunoblotting assays were carried out in serum and colostrum samples from puerperal women. Data are expressed as range of frequencies (%). *A*: All antigenic fractions detected in samples. Molecular weights (kDa) are shown at left side. Different colors represent different pixel density

Immunoblotting assays showed that it is possible to detect IgG, IgM and IgA antibodies specific to different antigen fractions of *T. gondii* in human serum and colostrum samples (Fig. 4). Anti-*T. gondii* IgG present in serum and colostrum samples recognized a higher number of antigenic fractions than the IgM and IgA antibodies specific for the parasite (Fig. 5). Furthermore, we observed that IgG antibodies reacted similarly to at least 20 antigenic fractions (97, 83, 66, 60, 57, 54, 50, 44, 38, 34, 32, 30, 29, 28, 26, 25, 24, 23, 22 and 21 kDa) in more than 75 % of serum samples. It is different from what was observed in colostrum samples, where recognition varied highly among patients with reactivity to at least 14 antigenic fractions (70, 50, 39, 34, 32, 29, 27, 26, 25, 24, 23, 20, 19 and 15 kDa), with less than 50 % of frequency (Figs. 5a and 6). However, considering paired serum and colostrum samples from the same patient, it was possible to observe that IgG present in the serum always recognized more *T. gondii* antigens than IgG antibodies present in colostrum.

Regarding IgM antibodies, a pattern of recognition of *T. gondii* antigenic fractions was observed only in serum samples, where more than 75 % of the samples reacted with three fractions (70, 54 and 30 kDa) (Figs. 5b and 6). The majority of fractions were detected with less than 50 % of frequency for both types of samples. Furthermore, when both biological samples from the same woman were taken into account, the IgM recognition profile of antigens also varied.

By analyzing IgA isotype, the profile of reactivity was opposite to the IgG antibodies. Considering both samples from the same patient, the colostrum IgA specific antibodies recognized a higher number of antigenic fractions than the IgA antibodies present in serum. Also, 97, 83 and 70 kDa fractions were recognized in more than 75 % of colostrum samples. However, no similar recognition pattern was observed in colostrum or serum samples from many puerperal women, when analyzed separately, since most antigenic fractions were recognized with frequencies below 50 % (Figs. 5c and 6).

### Avidity of IgG antibodies to antigenic fractions of *T. gondii*

**Fig. 7:**
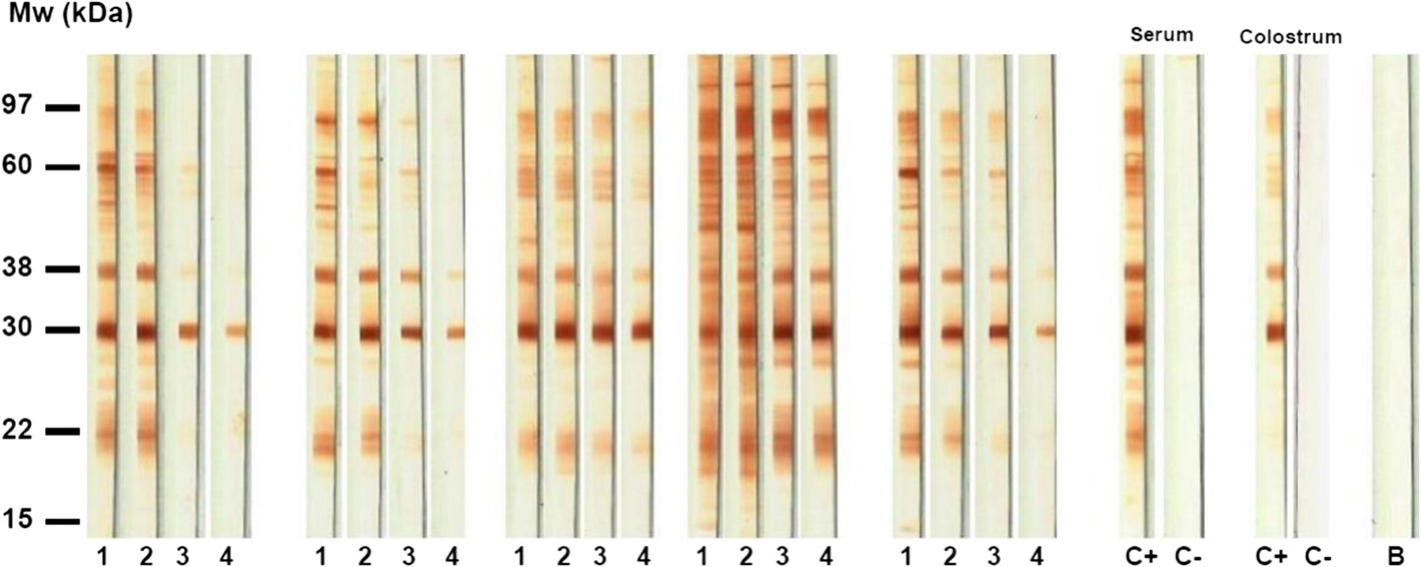
IgG avidity immunoblotting towards antigenic fractions of *T. gondii.* Representative immunoblotting assay to detect IgG avidity in serum and colostrum samples directed to soluble antigens of *T. gondii*. Molecular weights (kDa) are shown at left side. **1** and **2**: membrane strips incubated with serum sample and washed with untreated or 6 M urea-treated preparation, respectively. **3** and **4**: membrane strips incubated with colostrum sample and washed with untreated or 6 M urea-treated preparation, respectively. **C+** positive control. **C-** negative control. **B** blank

**Fig. 8:**
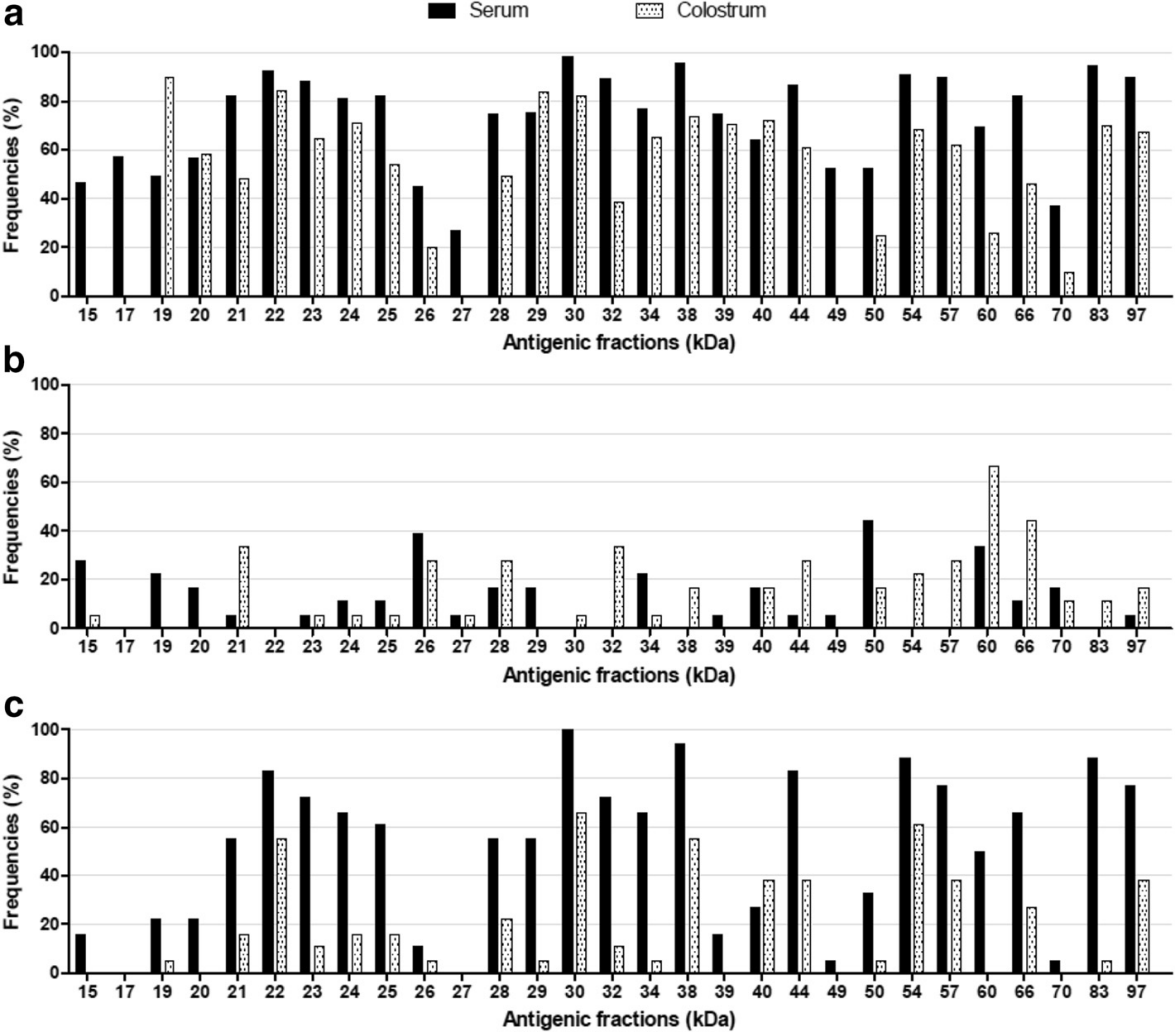
IgG avidity indices against *T. gondii* in serum and colostrum samples. The results are expressed in avidity index percentage (AI) towards *T. gondii* antigenic fractions. **a**. Average IgG avidity against each antigenic fraction. **b**. Frequencies from antigenic fractions recognized with low avidity (AI < 50 %). **c**. Frequencies from antigenic fractions recognized with high avidity (AI > 75 %)

As shown in Fig. 7, the immunoblotting assay to evaluate avidity of IgG antibodies was carried out with serum and colostrum samples from 21 out of 30 from puerperal patients, including from the risk group. Figure 8 shows the average avidity of IgG antibodies specific for each antigenic fractions of *T. gondii* (Fig. 8a), as well as the frequency of recognition for each fraction by IgG antibodies of low avidity (Fig. 8b) or high avidity (Fig. 8c).

All antigenic fractions detected with high frequency (>75 % samples) by serum IgG antibodies (Fig. 5a) are recognized by antibodies with high avidity, except for the 50 and 60 kDa fractions, with 50 % of patients having IgG antibodies with high avidity specific for them (Fig. 8c). In addition, no patient had IgG antibodies with low avidity in the serum in 22, 30, 32, 38, 54, 57 and 83 kDa fractions (Fig. 8b). Colostrum IgG antibodies showed a lower avidity in almost all antigenic fractions comparing with the avidity of serum IgG antibodies (Figs. 7 and 8a). Also, 60 and 66 kDa fractions were frequently recognized by colostrum IgG antibodies of low avidity (Fig. 8b). However, IgG antibodies of high avidity specific to 60 kDa fraction were not detected (Fig. 8c). On the other hand, 22, 30, 38 and 54 kDa fractions were often recognized by IgG antibodies of high avidity, for both types of biological samples (Fig. 8c).

### Clinical assistance and follow-up

During samples collection all patients received guidelines on the importance of breastfeeding, as well as how to breastfeed and how to hand milk to relieve engorged breast. In addition, the patients whose immunoassays of IgM and/or IgA were positive for *T. gondii* were informed, and their children had a follow-up appointment scheduled with a neonatologist at the Clinical Hospital, UFU. For these newborns, the following tests were carried out: serology for *T. gondii* (IgG and IgM), cranial ultrasonography to evaluate cerebral calcifications and ventriculomegaly as a sign of hydrocephalus, and funduscopy examination to evaluate chorioretinitis. All examinations were performed at the Clinical Hospital, UFU.

Nine patients did not attend the follow-up appointment and their children were not evaluated. The other newborns were examined and followed up for at least six months after birth to monitor the possibility of congenital infection. Funduscopy examination and ultrasonography of the skull showed normal results for most of them, together with negative IgM and decay of IgG level, indicating that it was the maternal antibody which was transferred. However, as shown in Table 2, three newborns manifested the disease with monitoring and treatment of the following cases:

**Table 2:** Summary of the three clinical cases from newborns diagnosed with congenital toxoplasmosis among the 289 puerperal women enrolled in the present study

Case number	Gestation month when mother was diagnosed with toxoplasmosis at prenatal care	Toxoplasmosis treatment during pregnancy	Cranial ultrasonography of neonate	Fundoscopy of neonate	Neonate serology for *T. gondii*		Serology when mother was at puerperal stage					
					IgG	IgM	Serum sample			Colostrum sample		
							IgG (EI)	IgM (EI)	IgA (EI)	IgG (EI)	IgM (EI)	IgA (EI)
1	5^th^	Yes	Dilatation of the lateral ventricles	Chorioretinitis	Pos	Pos	Pos (3.10)	Pos (3.59)	Pos (1.46)	Pos (7.32)	Pos (1.68)	Neg (0.58)
2	6^th^	Yes	Multiple cerebral calcifications	Chorioretinitis	Pos	Neg	Pos (4.76)	Pos (3.36)	Neg (1.01)	Pos (10.45)	Pos (3.11)	Neg (0.81)
3	Immune^a^	No	Normal	Chorioretinitis	Pos	Neg	Pos (4.82)	Pos (2.29)	Pos (2.77)	Pos (13.82)	Pos (1.24)	Pos (1.30)

^a^During prenatal care the mother was diagnosed as immune to toxoplasmosis

*Pos* Positive, *Neg* Negative, *EI* ELISA index

- Case 1 – During prenatal care the mother was diagnosed with acute toxoplasmosis in the second trimester and treated with sulfadiazine, pyrimethamine and folinic acid until the 34th week of pregnancy, and with spiramycin until delivery. Neonate presented positive IgM and IgG, dilatation of the lateral ventricles of the brain and abnormal fundoscopy, which characterizes hydrocephalus and chorioretinitis due to congenital toxoplasmosis infection.
- Case 2 – During prenatal care the mother was diagnosed with acute toxoplasmosis in the second trimester and treated with spiramycin until the end of pregnancy. Neonate presented negative IgM and positive IgG, multiple cerebral calcifications and chorioretinitis due to congenital toxoplasmosis infection.
- Case 3 – Neonate presented negative IgM and increased IgG, normal cranial ultrasonography, but eye damage diagnosed as chorioretinitis due to congenital toxoplasmosis. This case deserves emphasis because during prenatal care the mother was diagnosed as immune to toxoplasmosis, as only IgG antibodies were detected at that time. However, our results showed that in fact she had acute toxoplasmosis in the first days after delivery, which was confirmed by the detection of IgM and IgA specific to the parasite in serum and colostrum samples (Table 2).

## Discussion

It is generally accepted that *T. gondii* infection may cause severe fetal diseases, when occurring during pregnancy [2]. This vertical transmission may vary from 6 to 72 %, depending on the gestational period when it occurs, with the risk of transmission increasing from the 1^st^ to the 3^rd^ trimesters in human pregnancy. Among the serious fetal damages, when the maternal infection takes place during the 1^st^ trimester of pregnancy, are: decreased eyesight or blindness, hearing impairment or deafness and mental problems [17].

*Toxoplasma gondii* congenital infections are usually neglected because they are frequently asymptomatic at birth and may remain overlooked until months or years after birth [18], when they can emerge, usually as severe chorioretinitis [19]. Hence, it is extremely important to exclude the possibility of *T. gondii* infection even at puerperal period, since the mother may be asymptomatically infected during pregnancy. In the present study, it was demonstrated that in one of the cases of congenital toxoplasmosis, the pregnant woman was diagnosed as immune to toxoplasmosis, but actually she had an acute infection with *T. gondii* and her baby had severe ocular manifestation of the disease.

The production of IgG antibodies is important to control *T. gondii* infection, but IgG-producing plasma cells from breast tissue contribute little to the level of IgG in colostrum, when compared to IgG derived from serum [20, 21]. In the present study, a significant association between serum and colostrum IgG levels was observed, indicating that the majority of IgG isotype was transferred from plasma to mammary gland, explaining the immunoblotting assay results, which showed that serum IgG recognized more *T. gondii* antigenic fractions than colostrum IgG. In contrast, when the levels of IgA isotype were compared between serum and colostrum samples, a weak association was found. Furthermore, the immunoblotting assays showed that IgA antibodies present in colostrum samples recognized a higher number of antigenic fractions than serum IgA. This is a new piece of information concerning immune response to *T. gondii*, even though it is in agreement with the literature, which shows that most IgA is synthesized locally by plasma cells in mammary gland [21].

Chardès and colleagues [22] demonstrated the presence of *T. gondii*-specific IgG, IgM and IgA antibody in serum, intestinal secretions and milk from mice experimentally infected with cysts of the parasite via oral. They also showed that IgG antibodies present in serum and milk recognized more antigenic fractions than IgA and IgM isotypes, and IgG antibodies also detected most of the antigens recognized by IgA. This is in accordance with our results for human serum and colostrum samples described in the present study. However, it is necessary to make clear that there are limitations of this approach, taking into account the fact that the recognition of parasite antigenic fractions by different antibody isotypes depends on the individual immune response of the patients.

Although 39 kDa antigenic fraction was recognized by IgG and IgA serum and colostrum antibodies in less than 25 % if the samples, this fraction was recognized by IgG serum from all three puerperal mothers whose newborns presented clinically confirmed congenital toxoplasmosis. In addition, this fraction was also detected by colostrum IgA from two of these puerperal women. Thus, these results justify particular attention to determine the potential role of the 39 kDa *T. gondii* fraction as a suitable marker to diagnose congenital toxoplasmosis. Also, the 60 kDa fraction may be considered a good marker for acute toxoplasmosis infection because it was recognized frequently by IgG antibodies of low avidity, mainly in colostrum samples, including from mothers whose neonates presented vertical infection.

It is known that maternal IgG antibodies play a protective role in congenital toxoplasmosis, by reducing placental parasitemia, as well as by their transference to the fetus through the placenta [18, 23–26]. Our results showed that colostrum had a high level of specific IgG antibodies against *T. gondii* and, therefore, this may be a source of potentially protective IgG antibodies to minimize the damage of congenital infection in the newborn. In agreement with data from literature demonstrating that IgA isotype may remain biologically active on mucosal surfaces due to its proteolytic digestion resistance [27–30], the present study also demonstrated that human colostrum samples have high levels of IgA antibodies against *T. gondii*, providing effective protection to the child.

## Conclusions

The present study demonstrated that it is important to investigate the occurrence of congenital toxoplasmosis even at puerperal period, as we showed that *T. gondii-*specific IgG, IgM and IgA antibodies may be detected with significant correlations in serum and colostrum samples from puerperal women. These results suggest that colostrum may also be used as an alternative biological sample to efficiently diagnose congenital toxoplasmosis and that further studies are necessary to access if the colostrum immunoglobulins specific to *T. gondii* play a significant role in the control of congenital toxoplasmosis.

## Abbreviations

ABTS: 2,2′- azinobis-3-ethyl-benzthiazoline sulfonic acid
BCA: Bicinchoninic acid
DAB: adding diaminobenzidine
EI: ELISA index
ELISA: Enzyme Linked Immunosorbent Assay
HIV: immunodeficiency vírus
HTLV: Human T lymphotropic vírus
OD: optical density
PBS: phosphate buffered saline
PBS-T: PBS plus Tween 0.05 %
PBS-T-M1 %: PBS-T plus 1 % nonfat powdered milk
PBS-T-M5 %: PBS-T plus 5 % nonfat powdered milk
STAg: soluble tachyzoite antigen
